# Supplementary material for: Biasing Conformational Sampling in AlphaFold 3 and Boltz‑2 via Pair Representation Scaling
Source: J Chem Inf Model. 2026 Aug 19;66(17):10862–76. doi: 10.1021/acs.jcim.6c02094 (PMC13580555; doi:10.1021/acs.jcim.6c02094)
Supplement: Supplementary file 1 [file ci6c02094_si_001.pdf]

Supporting Information

Biasing Conformational Sampling in AlphaFold 3 and Boltz-2 via Pair Representation Scaling

Shosuke Suzuki[a] and Toshiyuki Amagasa[b]

[a]Graduate School of Science and Technology, University of Tsukuba, Tsukuba, 305-8573, Japan
[b]Center for Computational Sciences, University of Tsukuba, Tsukuba, 305-8577, Japan

CONTENTS

S1 Benchmark targets . . . S2
S2 Ensemble occupancy of the two states . . . S3
S3 Sample efficiency and reference-free selection . . . S3
S4 Distogram–experiment agreement across targets . . . S4
S5 Per-target supporting figures . . . S5
S5.1 Conformational sampling . . . S5
S5.2 Distogram bimodality . . . S6

S1 Benchmark targets

Table S1 lists the 86 benchmark targets. The source benchmarks are the domain-motion set of BioEmu [23], OC23 [9], IOMemP [42], and the entropy-guided folding membrane set (EGF) [13]. The BioEmu and OC23 targets form the domain-motion class and the IOMemP and EGF targets the transporter class. Six targets, Q5F9M1, Q18A65, A2RJ53, A0A075Q0W3, P33284 and P71447, belong to both the BioEmu and the OC23 set. They are listed once, under OC23, and their reference pairs are the ones BioEmu specifies, which is why the Methods count them among the 22 BioEmu proteins and take 17 further proteins from OC23. Difficulty is defined per predictor on that predictor's own default ensemble, binning its worst-case minimum Cα RMSD as easy (< 2 Å), medium (2–4 Å), or hard (≥ 4 Å), so a target can be easy for one predictor and hard for the other. The UniProt accessions, protein names and release dates were read from the RCSB Protein Data Bank.

Table S1. The 86 benchmark targets. UniProt accession, protein, source benchmark, the two reference structures with their author chains and Protein Data Bank release dates, and the difficulty assignment for each predictor. For 4 targets the two references come from different organisms or strains of the same protein, and both accessions are listed.

UniProt	Protein	Set	Ref. 1	Released	Ref. 2	Released	AlphaFold 3	Boltz-2
A0A0H3AFX3	GGDEF and EAL domain-containing p…	BioEmu	6PWJ (A)	2019-10-09	6PWK (A)	2019-10-09	Easy	Easy
B0F0C5	Putative DNA-mediated transposase	BioEmu	6PQN (A)	2019-10-09	6PQX (A)	2019-10-09	Hard	Hard
P02925	D-ribose-binding protein	BioEmu	1URP (A)	1998-06-17	2DRI (A)	1995-01-26	Medium	Easy
P02911	Lysine/arginine/ornithine-binding…	BioEmu	6ML0 (A)	2019-08-07	6MLP (E)	2019-08-07	Hard	Hard
P03047	Antitermination protein Q	BioEmu	7UBJ (A)	2022-09-28	7UBL (A)	2022-09-28	Hard	Hard
P06766	DNA polymerase beta	BioEmu	1BPD (A)	1994-06-22	2BPG (A)	1994-08-31	Hard	Hard
P0A8W0	HTH-type transcriptional represso…	BioEmu	6ON4 (A)	2020-07-08	6WFQ (C)	2021-03-10	Hard	Medium
P0AEQ3	Glutamine binding protein	BioEmu	1GGG (A)	1996-11-08	1WDN (A)	1998-05-06	Easy	Easy
P0DP23	Calmodulin	BioEmu	1CLL (A)	1993-10-31	5GGM (A)	2016-09-28	Easy	Hard
P23847	Dipeptide-binding protein	BioEmu	1DPE (A)	1996-08-17	1DPP (A)	1995-12-07	Easy	Medium
P24182	Biotin carboxylase	BioEmu	1DV1 (A)	2000-06-09	1DV2 (A)	2000-06-09	Easy	Easy
P28366	Preprotein translocase secA	BioEmu	1M74 (A)	2002-09-20	1TF2 (A)	2004-08-03	Medium	Hard
P37487	Manganese-dependent inorganic pyr…	BioEmu	1K23 (C)	2001-10-31	1WPM (B)	2004-11-23	Easy	Medium
P67701	Antitoxin HigA	BioEmu	6JQ4 (A)	2019-05-01	6KML (B)	2020-11-18	Hard	Hard
P69441	Adenylate kinase	BioEmu	1AKE (A)	1994-01-31	4AKE (A)	1996-06-10	Medium	Easy
Q83VS8	EcoT38I restriction endonuclease	BioEmu	7E8R (A)	2022-03-02	7EDB (A)	2022-03-16	Hard	Hard
A0A075Q0W3	penicillin binding protein 5 (PBP…	OC23	6MKA (A)	2018-10-31	6MKJ (A)	2018-10-31	Hard	Medium
A0QTT2	ABC1 family protein	OC23	7CY2 (A)	2021-12-08	7CYR (A)	2021-12-08	Medium	Medium
A2RJ53	Oligopeptide-binding protein oppA	OC23	3DRF (A)	2009-03-31	3FTO (A)	2009-03-31	Easy	Easy
A6UVT1	5,10-methenyltetrahydromethanopte…	OC23	6HAC (A)	2019-08-28	6HAE (A)	2019-08-28	Easy	Medium
B3EYN2	Extracellular arabinanase	OC23	5HO0 (A)	2017-02-01	5HO2 (A)	2017-02-01	Easy	Medium
B7IE18	Lipid II flippase MurJ	OC23	6NC6 (A)	2019-04-17	6NC7 (A)	2019-04-17	Medium	Medium
O76728	Oligopeptidase B	OC23	4BP8 (A)	2014-02-12	4BP9 (A)	2014-02-12	Hard	Hard
P00558	Phosphoglycerate kinase 1	OC23	2WZD (A)	2010-04-14	2XE6 (A)	2011-01-19	Hard	Easy
P21589	5'-nucleotidase	OC23	4H2I (A)	2012-11-28	7QGA (A)	2022-02-16	Medium	Medium
P31133	Putrescine-binding periplasmic pr…	OC23	6YE0 (A)	2021-01-20	6YED (A)	2021-01-20	Easy	Medium
P33284	Hexokinase	OC23	3O80 (A)	2010-10-13	3O8M (A)	2010-10-13	Easy	Easy
P40131	Flagella basal body P-ring format…	OC23	3TEE (A)	2012-08-15	3VJP (A)	2012-10-31	Hard	Easy
P62495	Eukaryotic peptide chain release…	OC23	2KTU (A)	2010-06-09	2KTV (A)	2010-06-09	Hard	Hard
P71447	Beta-phosphoglucomutase	OC23	2WF5 (A)	2009-09-15	2WFA (A)	2010-05-26	Easy	Easy
Q18A65	ABC-type transport system, sugar…	OC23	6HNK (A)	2019-08-28	6HNI (A)	2019-08-28	Hard	Easy
Q53W80	Sugar ABC transporter	OC23	7C63 (A)	2020-09-16	7C66 (A)	2020-09-16	Easy	Easy
Q5F9M1	Putative ABC transporter	OC23	2YLN (A)	2011-12-14	3ZSF (A)	2011-12-14	Hard	Easy
Q7DAU8	Betaine ABC transporter permease…	OC23	3L6G (A)	2010-05-19	3L6H (A)	2010-05-19	Easy	Easy
Q9ERE7	Mesoderm development candidate 2	OC23	2RQK (A)	2009-08-25	2RQM (A)	2009-08-25	Hard	Hard
Q9SS90	E3 ubiquitin-protein ligase RGLG1	OC23	6K85 (B)	2020-06-24	6K8B (A)	2020-06-24	Easy	Easy
Q9X6R4	Prolyl endopeptidase	OC23	3IUJ (A)	2010-05-05	3IUQ (A)	2010-05-05	Hard	Hard
Q9X9P9	nikD protein	OC23	2OLN (A)	2007-07-31	2OLO (A)	2007-07-31	Easy	Easy
Q9Z4N6	iron binding protein FbpA	OC23	1SI0 (A)	2004-06-08	1SI1 (A)	2004-06-08	Easy	Easy
J7QAK3	D-galactonate transport	IOMemP	6E9N (B)	2019-05-22	6E9O (B)	2019-05-22	Easy	Medium
P18238, G2QNH0	ADP, ATP carrier protein 3	IOMemP	4C9J (B)	2014-01-22	6GCI (A)	2019-01-09	Hard	Medium
M1VAN7	ATP-binding cassette, sub-family…	IOMemP	6A6N (A)	2019-01-16	7DQV (A)	2021-03-24	Hard	Medium
F1NCD6, Q9DA75	Major Facilitator Superfamily Dom…	IOMemP	7MJS (X)	2021-06-16	7N98 (A)	2021-08-04	Medium	Hard
Q8U2X0	Putative uncharacterized protein	IOMemP	3VVS (A)	2013-04-03	6FHZ (A)	2019-05-08	Hard	Hard
P43005	Excitatory amino acid transporter…	IOMemP	6S3Q (A)	2020-07-08	6X3E (A)	2021-03-17	Medium	Easy
Q9WYC3	ABC transporter, ATP-binding prot…	IOMemP	3QF4 (A)	2012-03-28	6QV1 (C)	2019-05-29	Hard	Hard
Q9WYC4	Uncharacterized ABC transporter A…	IOMemP	4Q4A (B)	2014-07-16	6QV1 (B)	2019-05-29	Hard	Hard
Q72J04	Multidrug resistance ABC transpor…	IOMemP	5MKK (B)	2017-01-18	6RAJ (B)	2019-07-31	Hard	Medium
P0AEY8	Major Facilitator Superfamily mul…	IOMemP	6GV1 (A)	2018-10-03	6VS1 (A)	2020-06-03	Hard	Hard
P30878	Melibiose carrier protein	IOMemP	4M64 (A)	2014-01-15	7L17 (A)	2021-08-04	Hard	Hard
B7IE18	Lipid II flippase MurJ	IOMemP	6NC7 (A)	2019-04-17	6NC9 (A)	2019-04-17	Hard	Medium
Q63GK8	Protein-N(Pi)-phosphohistidine-su…	IOMemP	5IWS (A)	2016-05-25	6BVG (A)	2018-05-23	Easy	Hard

Continued on the next page.

Table S1 continued from the previous page.

UniProt	Protein	Set	Ref. 1	Released	Ref. 2	Released	AlphaFold 3	Boltz-2
Q6MLJ0	Solute carrier family 39 (Iron-re . . .	IOMemP	5AYM (A)	2015-11-04	6BTX (A)	2018-09-19	Easy	Easy
O86150	ATP-driven flippase PglK	IOMemP	5C78 (A)	2015-08-19	6HRC (D)	2019-03-06	Hard	Hard
Q14973	Sodium/bile acid cotransporter	EGF	7PQG (A)	2022-05-18	7VAG (A)	2022-05-25	Medium	Easy
Q72IM4, Q5SIA2	Na(+)/H(+) antiporter	EGF	4BWZ (A)	2013-08-28	5BZ2 (A)	2016-01-20	Easy	Easy
O59010	Glutamate transporter homolog	EGF	6BMI (B)	2018-01-17	6X16 (C)	2020-11-18	Hard	Easy
P0AGF4	D-xylose-proton symporter	EGF	4JA4 (C)	2013-05-01	6N3I (A)	2019-03-27	Medium	Medium
Q57837	Putative zinc metalloprotease MJ0 . . .	EGF	3B4R (A)	2008-01-15	3B4R (B)	2008-01-15	Hard	Medium
P09391	Protein GlpG	EGF	2NRF (A)	2006-11-14	6PJU (A)	2019-11-27	Medium	Medium
A0A380PV03	Transporter, sodium/bile acid sym . . .	EGF	6LH1 (A)	2020-12-09	7CYK (B)	2021-01-13	Medium	Medium
D6R8X8	Hydantoin permease	EGF	2X79 (A)	2010-05-05	4D1C (A)	2014-07-02	Medium	Medium
O67854	Na(+):neurotransmitter symporter . . .	EGF	6XWM (A)	2020-05-06	7DIX (A)	2021-04-07	Medium	Medium
G4CRQ5	Nucleoside permease	EGF	5L2B (A)	2017-04-12	5L2B (C)	2017-04-12	Easy	Hard
P02920	Lactose permease	EGF	2Y5Y (A)	2011-06-15	5GXB (A)	2016-10-26	Easy	Medium
P58353, P43427	Solute carrier family 2, facilita . . .	EGF	4YB9 (D)	2015-10-14	4YBQ (A)	2015-10-07	Easy	Medium
P53985	Monocarboxylate transporter 1	EGF	7CKO (A)	2020-12-23	7CKR (A)	2020-12-23	Easy	Easy
P31645	Sodium-dependent serotonin transp . . .	EGF	6DZZ (A)	2019-04-24	7TXT (S)	2023-03-15	Medium	Medium
O60931	Isoform 2 of Cystinosin	EGF	8DKX (P)	2022-09-21	8DYP (A)	2022-11-16	Easy	Medium
Q15758	Neutral amino acid transporter B(. . .	EGF	8OUJ (B)	2024-05-01	8OUJ (C)	2024-05-01	Hard	Medium
P13866	Sodium/glucose cotransporter 1	EGF	7SLA (A)	2021-12-15	7WMV (A)	2022-11-16	Medium	Medium
Q9LT15	Sugar transport protein 10	EGF	6H7D (A)	2019-02-06	7AAR (A)	2021-08-18	Medium	Medium
Q8IVW8	Sphingosine-1-phosphate transport . . .	EGF	8EX6 (A)	2023-05-31	8EX7 (A)	2023-05-31	Easy	Easy
Q9LFP6	Auxin efflux carrier component 8	EGF	7QP9 (B)	2022-07-06	7QPC (B)	2022-07-06	Easy	Easy
Q91X85	Feline leukemia virus subgroup C . . .	EGF	8VZN (A)	2024-05-01	8VZO (A)	2024-05-01	Easy	Easy
Q9NS82	Asc-type amino acid transporter 1	EGF	8WNT (B)	2024-05-15	8WNY (B)	2024-05-15	Medium	Medium
Q9GZV3	High affinity choline transporter . . .	EGF	8J74 (A)	2024-04-03	8J76 (A)	2024-04-10	Medium	Medium
P48067	Sodium- and chloride-dependent gl . . .	EGF	6ZPL (A)	2021-03-17	8WFK (A)	2024-04-03	Easy	Easy
A0ACD6B946	Nitrate transport permease protein	EGF	8W9M (A)	2024-02-28	8WM7 (A)	2024-02-28	Medium	Medium
Q05940	Synaptic vesicular amine transpor . . .	EGF	8JT5 (A)	2024-05-08	8XO9 (A)	2024-05-08	Easy	Medium
P54219	Chromaffin granule amine transpor . . .	EGF	8TGG (B)	2024-03-20	8TGI (A)	2024-03-20	Medium	Easy
Q8N697	SLC15A4-TSLAA/EGPF tag protein	EGF	8JZU (A)	2023-09-27	8P6A (A)	2023-06-07	Easy	Easy
O15245	Solute carrier family 22 member 1	EGF	8JTZ (A)	2024-03-27	8SC3 (A)	2023-10-18	Medium	Medium
Q84TH5	ABC transporter G family member 25	EGF	8I3B (B)	2023-09-13	8WBX (B)	2023-09-27	Medium	Medium
Q8NEW0	Zinc transporter 7	EGF	8J7W (B)	2023-09-20	8J7X (B)	2023-09-20	Hard	Hard
Q9BZW2	Solute carrier family 13 member 1	EGF	8W6T (A)	2024-04-17	8W6T (B)	2024-04-17	Hard	Hard

S2 Ensemble occupancy of the two states

Occupancy of a state is the fraction of the 250-model ensemble within 2 Å Cα RMSD of that reference, measured here for every target and method (Fig. S1).

Occupancy of the harder state is low throughout. Under default inference the ensemble never reaches that state for 63% of the AlphaFold 3 targets and 66% of the Boltz-2 targets, and scaling lowers those fractions to 44% and 55% (Fig. S1A,B). Where the state is reached the occupancy stays small, with the per-target median across the domain-motion group rising from 0 to 0.052 in AlphaFold 3, or 13 of 250 models. Scaling raises the harder-state occupancy over default inference in all three AlphaFold 3 groups and in the Boltz-2 domain-motion and after-cutoff groups (paired Wilcoxon signed-rank test, $p \leq 0.046$), but not in the Boltz-2 transporter group ($p = 0.73$).

The gain comes with a shift away from the dominant state (Fig. S1C). Following the reference that the default ensemble covers better, the median occupancy of that state falls from 0.97 to 0.74 in AlphaFold 3 and from 0.86 to 0.73 in Boltz-2, and it decreases for 56 of the 72 and 56 of the 74 targets whose default ensemble reaches it at all. Scaling therefore redistributes ensemble mass between the two states rather than adding coverage of the alternative state on top of the dominant one.

The best-minimum TM-score rises under scaling in every group of both predictors, by 0.001 to 0.040, but the 95% bootstrap confidence intervals of the paired conditions overlap in all six, so it separates the conditions less sharply than the three metrics of Figure 1 (Fig. S1D).

S3 Sample efficiency and reference-free selection

We first asked whether the scaling parameter itself carries information about the conformational state (Fig. S3A). Within each target we measured the Spearman correlation between β and the difference in TM-score to the two reference structures across the scaled models. The correlation was moderate and significant for most targets, more strongly in AlphaFold 3 than in Boltz-2 (Fig. S3A). The sign of the effective direction was target-specific, positive for roughly half the targets and negative for the rest, so no single direction of β maps onto a given conformational state across targets.

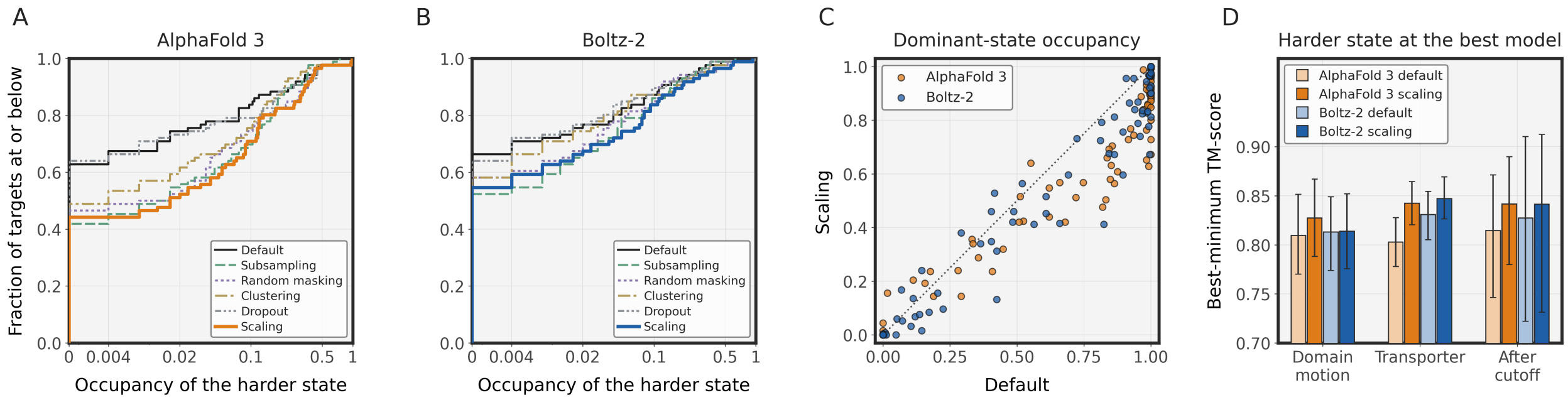

Figure S1. Ensemble occupancy of the two reference states. Occupancy is the fraction of the 250-model ensemble within 2 Å Cα RMSD of a reference state. **(A, B)** Distribution across targets of the occupancy of the harder state, as an empirical cumulative distribution, for AlphaFold 3 (A) and Boltz-2 (B). The value at zero occupancy is the fraction of targets whose ensemble never reaches that state. Scaling is drawn in the predictor colour and the baselines in the lighter tint. **(C)** Occupancy of the dominant state per target, default inference against scaling. The dominant state is the reference that the default ensemble covers better, and the same reference is followed into the scaled ensemble. The dotted line is the identity. **(D)** Best-minimum TM-score by conformational-change group, as target means with 95% bootstrap confidence intervals.

The broadening did not come from drawing more models. We resampled each method's ensemble at random, from a single model up to the full ensemble, and recomputed two-state per-state success at each size (Fig. S2A,B). Scaling reached the per-state coverage of the full default ensemble from a fraction of it, about a tenth in AlphaFold 3 and under half in Boltz-2, and stayed above every baseline across the whole range. The gain over default inference is therefore a property of the per-sample distribution rather than of the number of models drawn.

We then tested whether two models covering both conformational states could be selected from a scaled ensemble without a reference structure (Fig. S3B,C). A rule returned two models, scored by the area under a success curve, where success at a TM-score threshold means that the two models include one within that threshold of each reference structure. The per-target correlation between β and the state did not translate into a selection signal. Sign-based selection matched a random baseline for both predictors. Mean pLDDT peaked near default inference and declined with the magnitude of β, so the most confident models concentrated on the dominant state and selection by the two highest-pLDDT models fell below the random baseline. Structural clustering of the ensemble's secondary-structure-region Cα distance maps, returning the two cluster medoids, recovered both states more often than chance and approached the oracle ceiling for both predictors (Fig. S3B,C). The scaled ensemble therefore holds both conformational states in a form that an unsupervised structural criterion can separate, whereas the predictor's confidence is biased toward the dominant state and the β sign, though correlated with the state, is too target-specific to act on without a reference.

Selection rules. The success curve underlying each rule varies the TM-score threshold from 0.5 to 1.0, and a target counts as recovered at a threshold when the two returned models include one within it of the first reference structure and one within it of the second. Sign-based selection draws one model at random from the models generated with $\beta < 0$ and one from those with $\beta > 0$. Confidence selection returns the two models of highest mean Cα pLDDT. Structural clustering partitions the ensemble into two groups by k-means with $k = 2$ on the Cα distance maps restricted to the consensus secondary-structure residues, those assigned to a helix or strand by DSSP in at least half of the models, and returns the medoid of each group. The random baseline draws two models uniformly, and the oracle is the best two-model coverage attainable from the ensemble with knowledge of the reference structures.

S4 Distogram–experiment agreement across targets

Figure S4 shows, for every AlphaFold 3 target with a recovery improvement, the in-range Pearson correlation between the distogram change under scaling and the experimental distance change between the two reference states. The three targets used in Figure 5E–G are marked. The agreement is positive for 34 of the 35 targets (median $r = 0.56$), so the examples are representative of the full set.

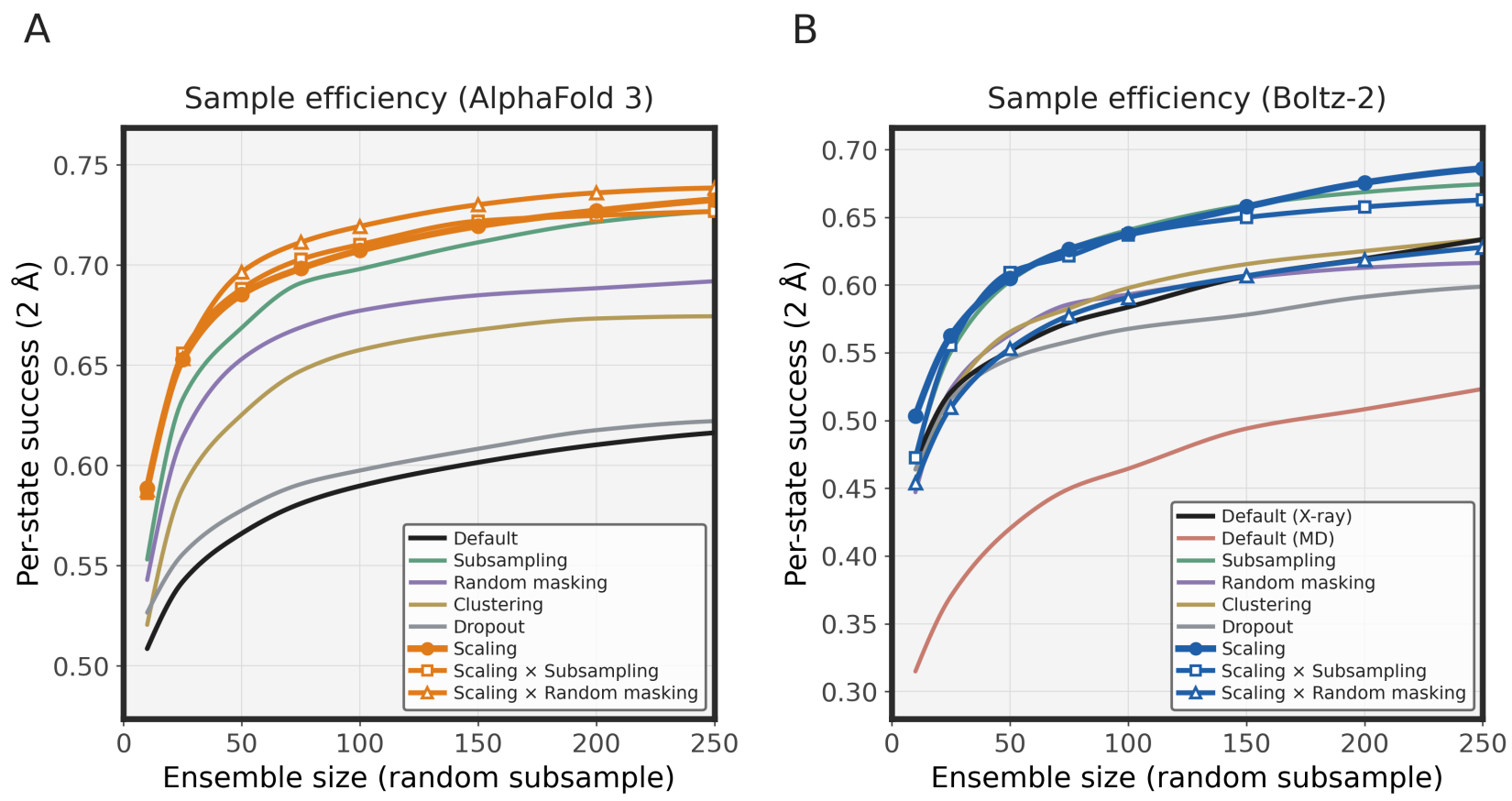

Figure S2. Sample efficiency. Per-state success at 2 Å as a function of ensemble size, drawn at random from the full ensemble, for **(A)** AlphaFold 3 and **(B)** Boltz-2. Default inference, the sampling baselines, scaling, and the two scaling combinations are all compared at equal ensemble sizes.

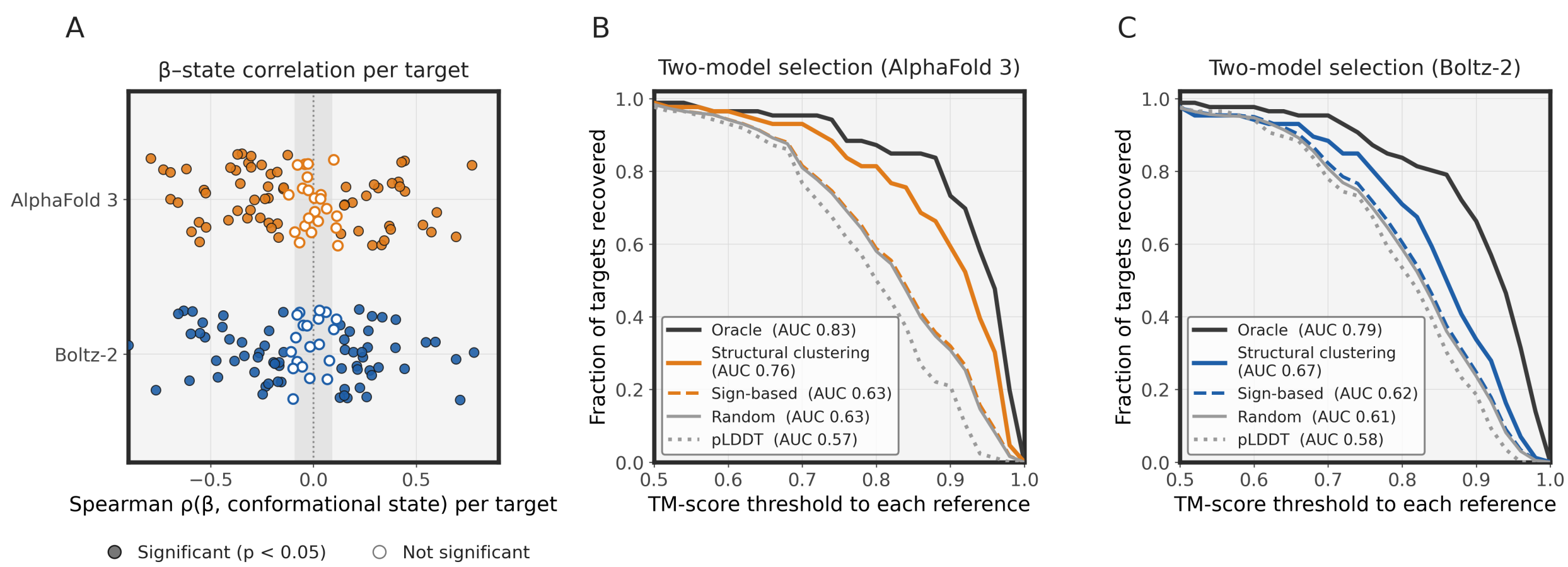

Figure S3. β–state correlation and reference-free model selection. (A) Per-target Spearman correlation between β and the TM-score difference to the two reference structures. Filled points are significant ($p < 0.05$), and the shaded band marks the non-significant range. **(B, C)** Reference-free selection of two models from the scaled ensemble for AlphaFold 3 (B) and Boltz-2 (C). Success at a TM-score threshold means the two selected models include one within that threshold of each reference. Rules: oracle, structural clustering of secondary-structure-region distance maps, sign-based selection, random baseline, and two highest-pLDDT models.

S5 Per-target supporting figures

S5.1 Conformational sampling

For every benchmark target, Figures S5–S10 resolve the conformational sampling of both predictors to individual models. Each target is shown as four panels, from left to right AlphaFold 3 under default inference, AlphaFold 3 under scaling, Boltz-2 under default inference, and Boltz-2 under scaling. Each panel plots the TM-score of every model to the first reference against its TM-score to the second, coloured by β, so the spread of an ensemble between the two states is visible directly. Targets are grouped by conformational-change class and the axis range is set per target.

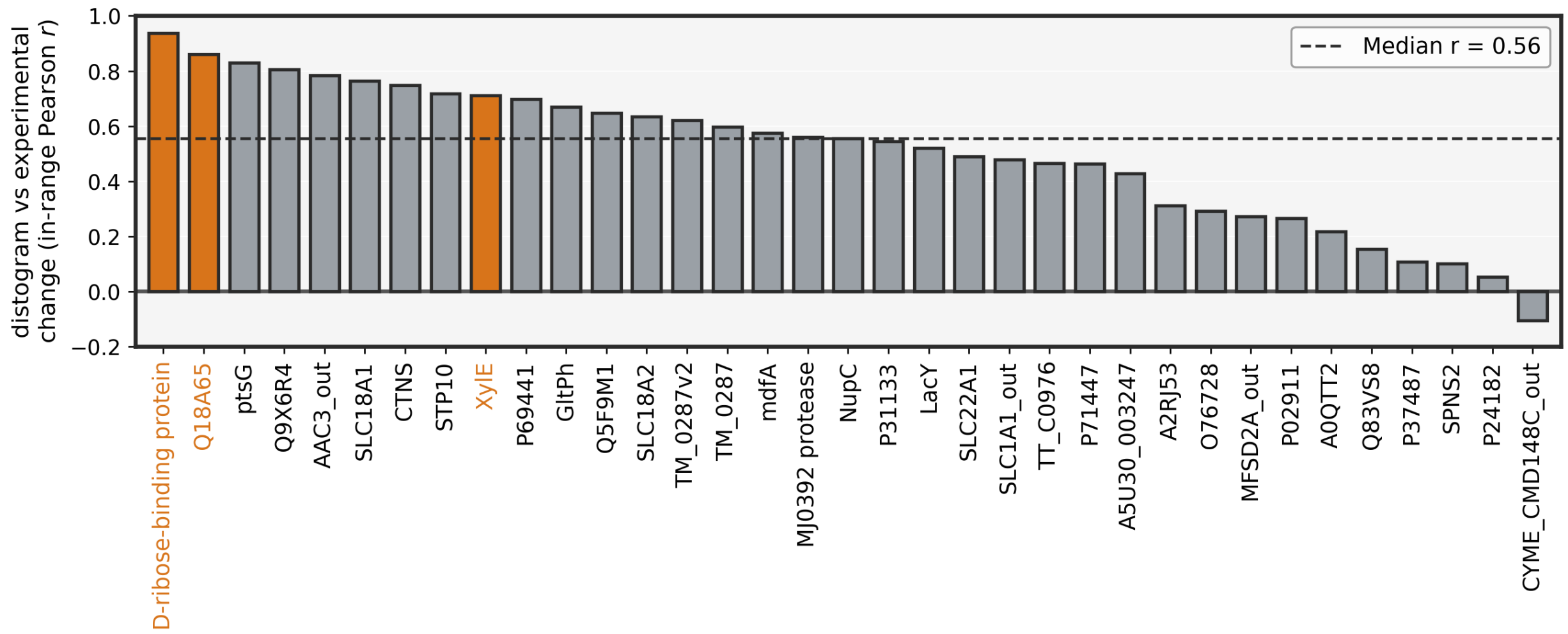

Figure S4. Distogram–experiment agreement for every AlphaFold 3 target with a recovery improvement. For each target, the bar is the in-range Pearson correlation between the per-residue-pair distogram change under scaling (at the target's $\beta^{\star}$) and the experimental distance change between its two reference structures. The three Figure 5 examples are highlighted. The dashed line is the median.

S5.2 Distogram bimodality

For each of the 86 benchmark targets, Figures S11–S16 show the AlphaFold 3 distogram at one representative change-contact pair across the 11 values of β. For each target the pair was chosen as the change-contact pair maximising the number of β values at which the pair is classified as bimodal with fitted modes matching both reference distances, with ties broken by the largest reference distance change. If no β value gives a matched bimodal classification the pair with the largest reference distance change is shown. Background colour encodes the classification at each β as in Figure 6C, pink for bimodal with peaks matching both reference distances, lavender for bimodal but unmatched, and grey for unimodal.

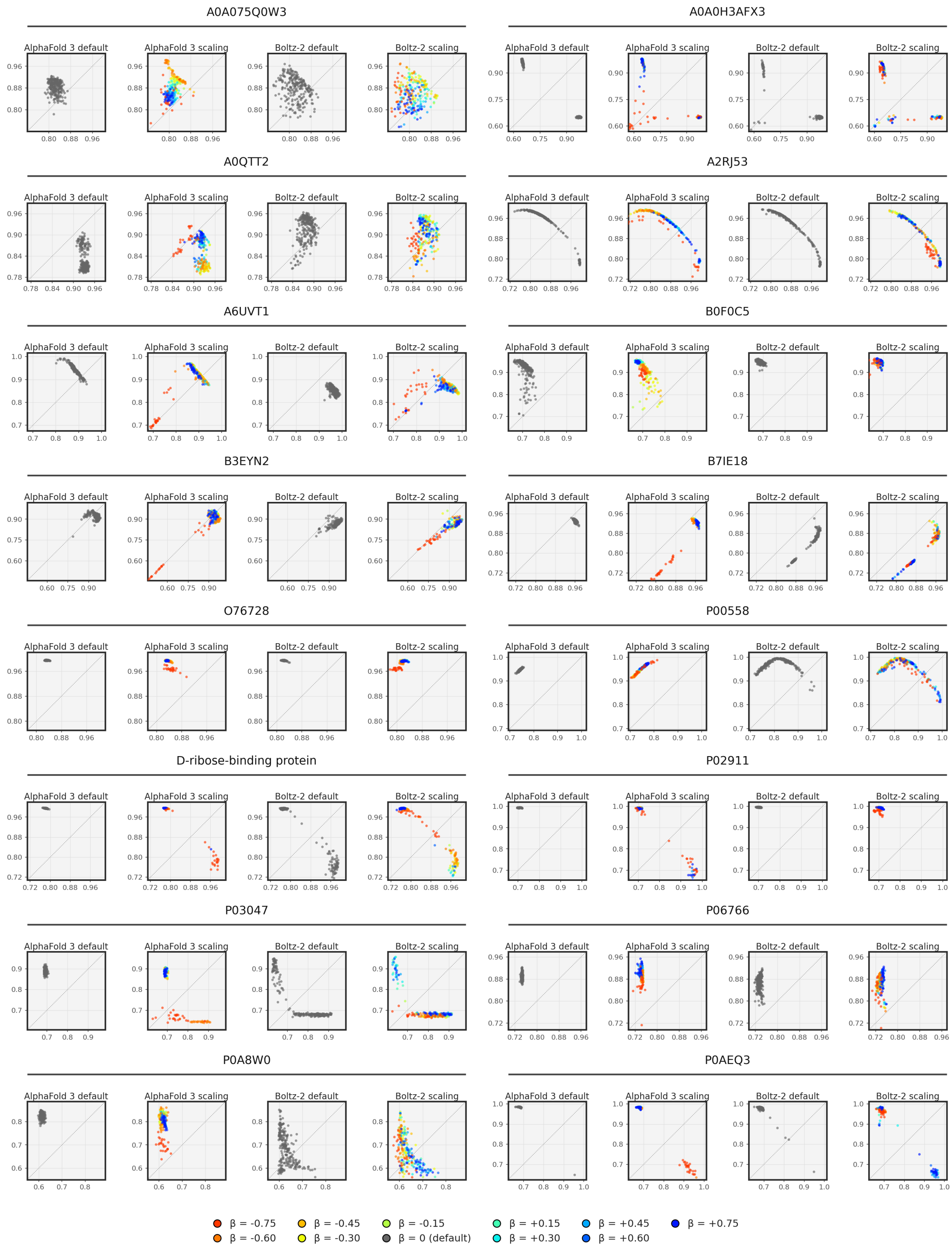

Figure S5. Per-target TM-score scatter, 1 of 6 (domain-motion targets). Each target is shown as four panels, namely AlphaFold 3 and Boltz-2 under default inference and under pair representation scaling, of the TM-score to reference 1 against the TM-score to reference 2, with points coloured by β ($\beta = 0$ is default inference).

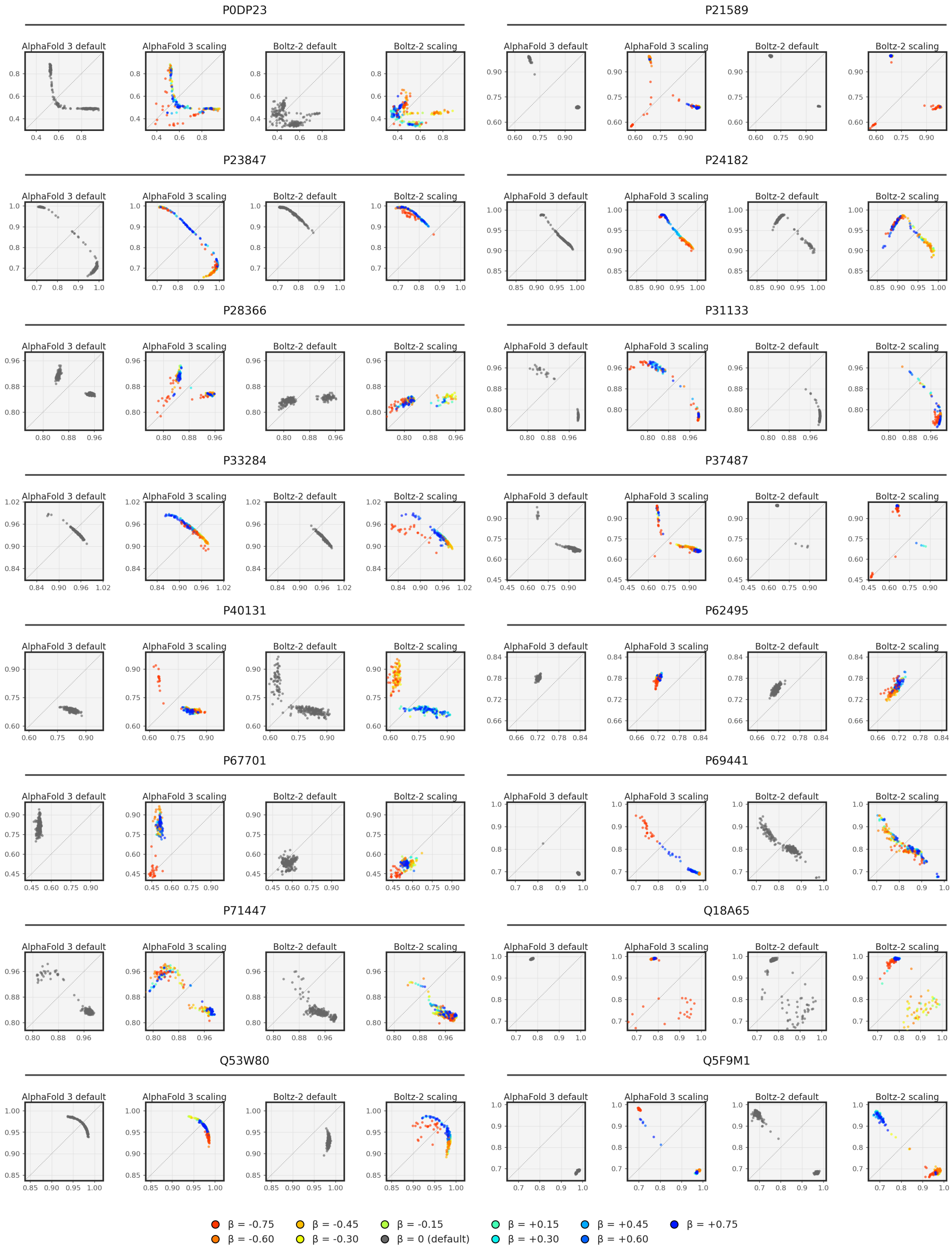

Figure S6. Per-target TM-score scatter, 2 of 6 (domain-motion targets). Panels as in Figure S5.

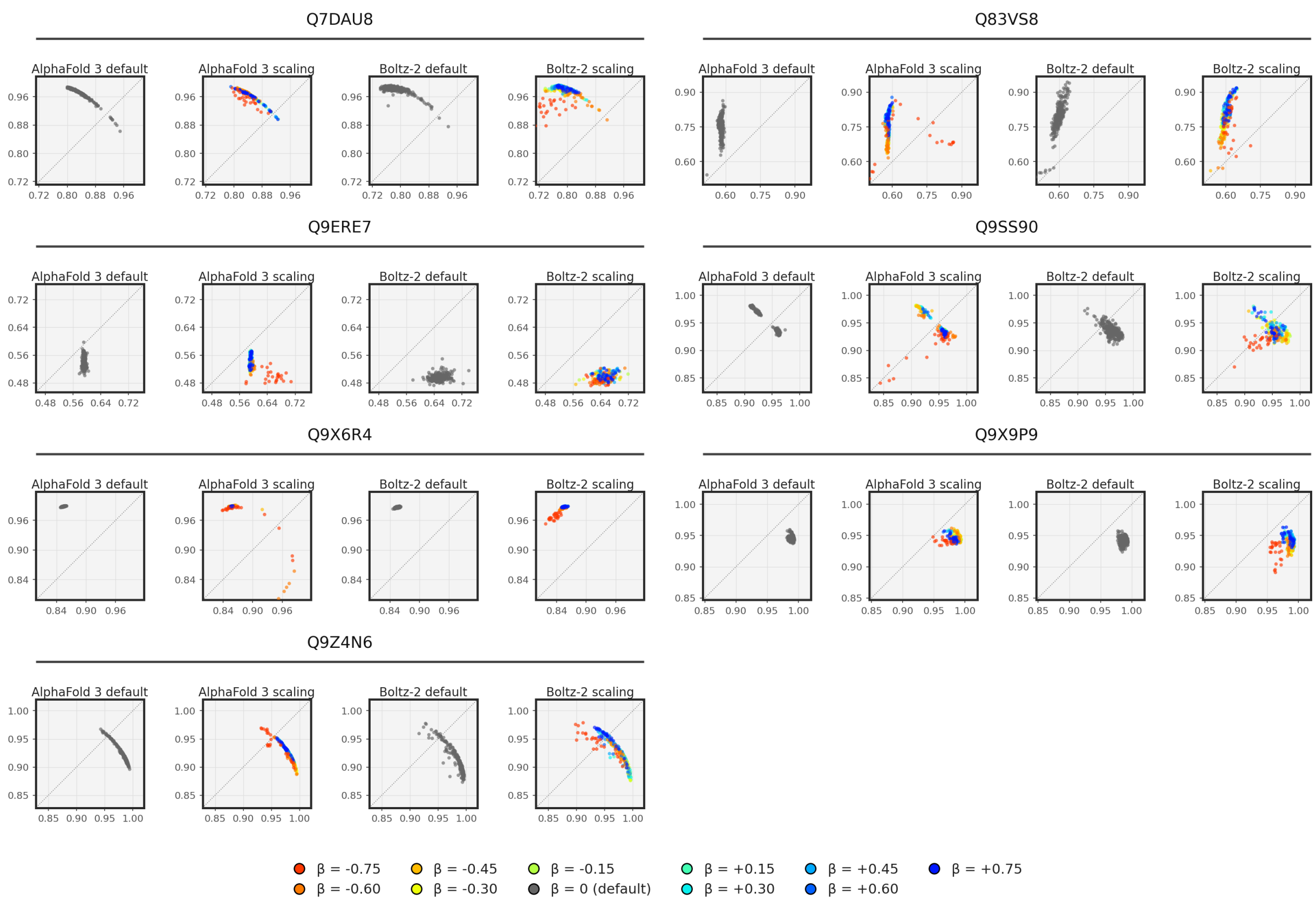

Figure S7. Per-target TM-score scatter, 3 of 6 (domain-motion targets). Panels as in Figure S5.

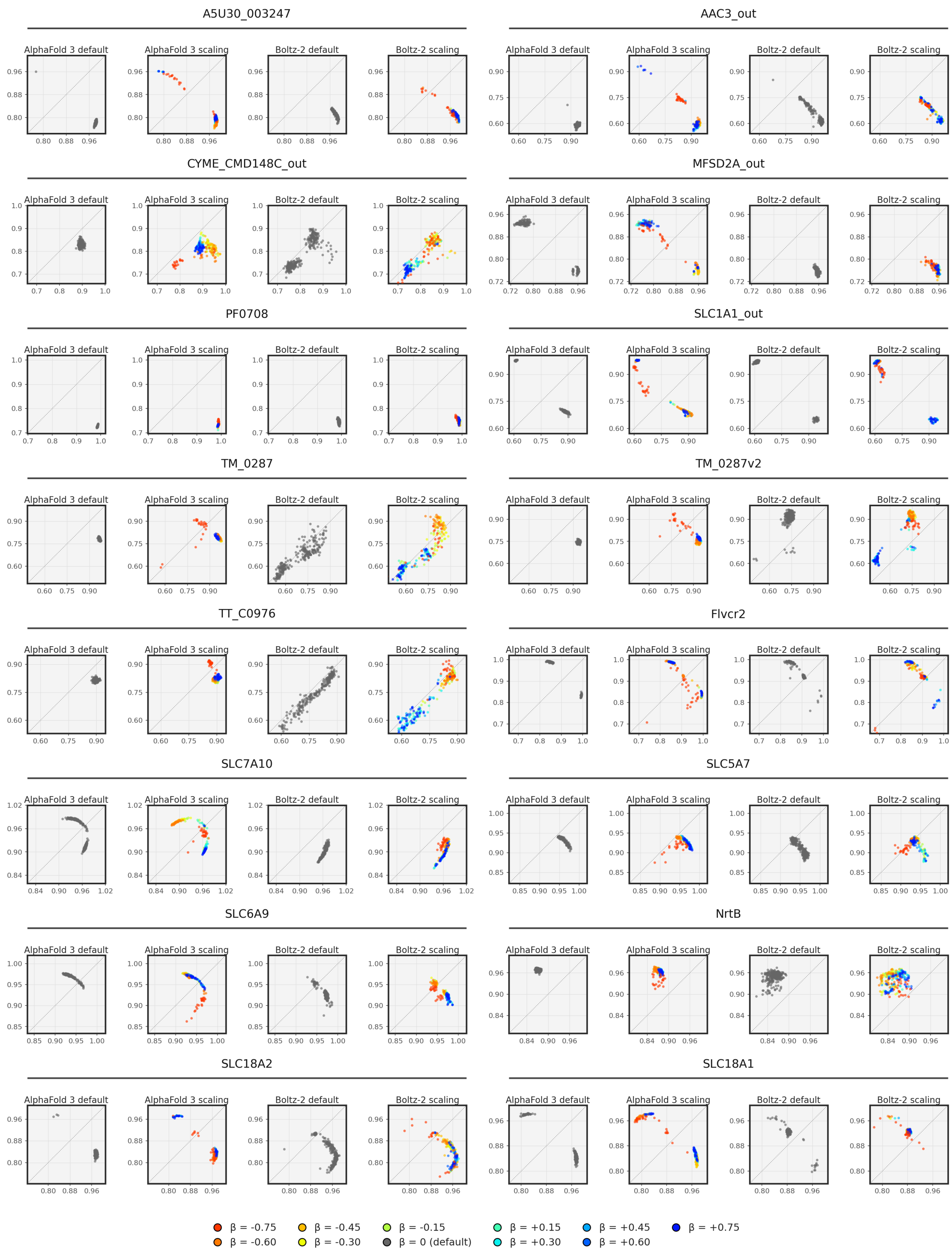

Figure S8. Per-target TM-score scatter, 4 of 6 (transporter targets). Panels as in Figure S5.

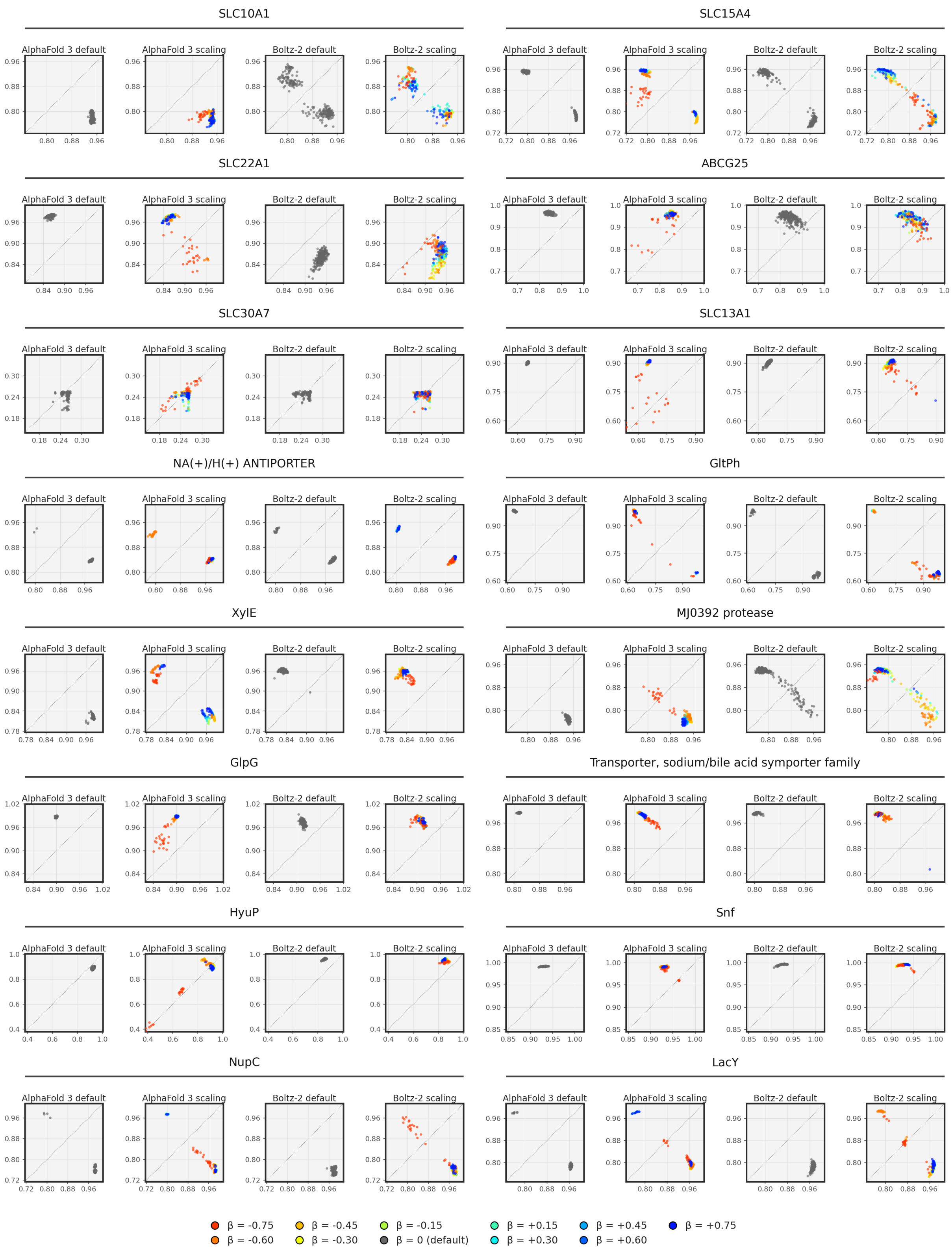

Figure S9. Per-target TM-score scatter, 5 of 6 (transporter targets). Panels as in Figure S5.

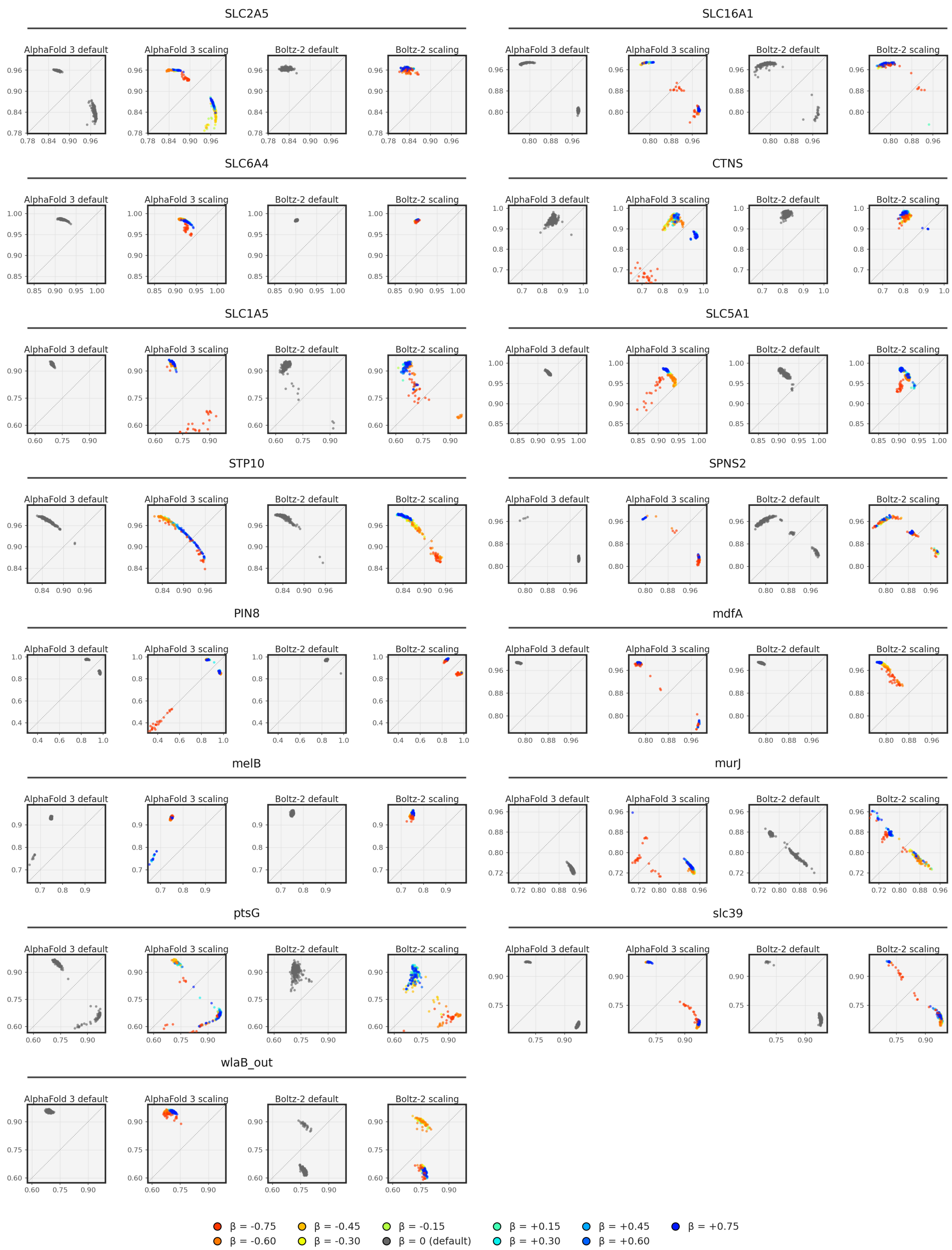

Figure S10. Per-target TM-score scatter, 6 of 6 (transporter targets). Panels as in Figure S5.

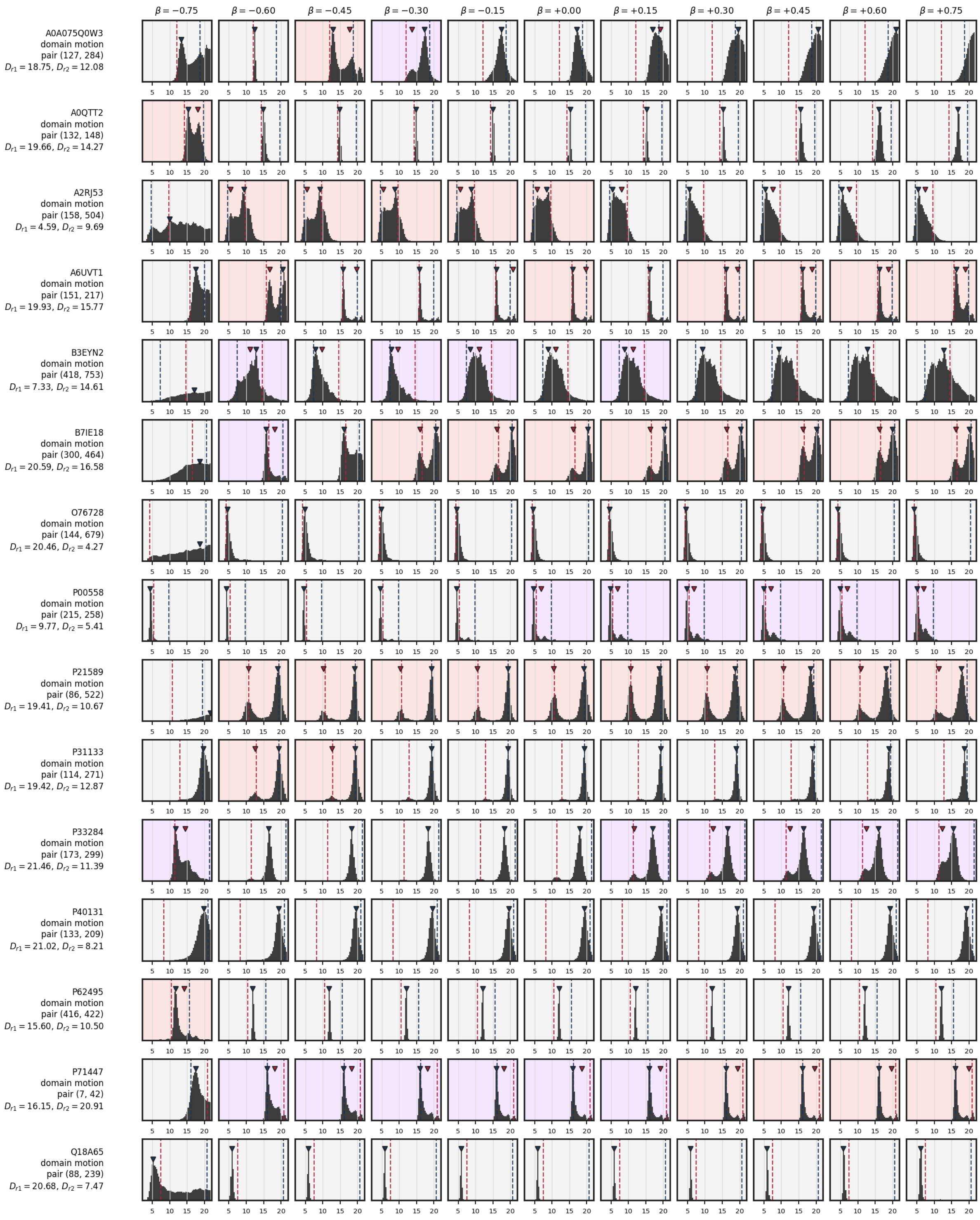

Figure S11. Per-target distogram bimodality, page 1 of 6. Each row is one target and shows the AlphaFold 3 distogram at the representative change-contact pair across the 11 values of β (selection criterion in text).

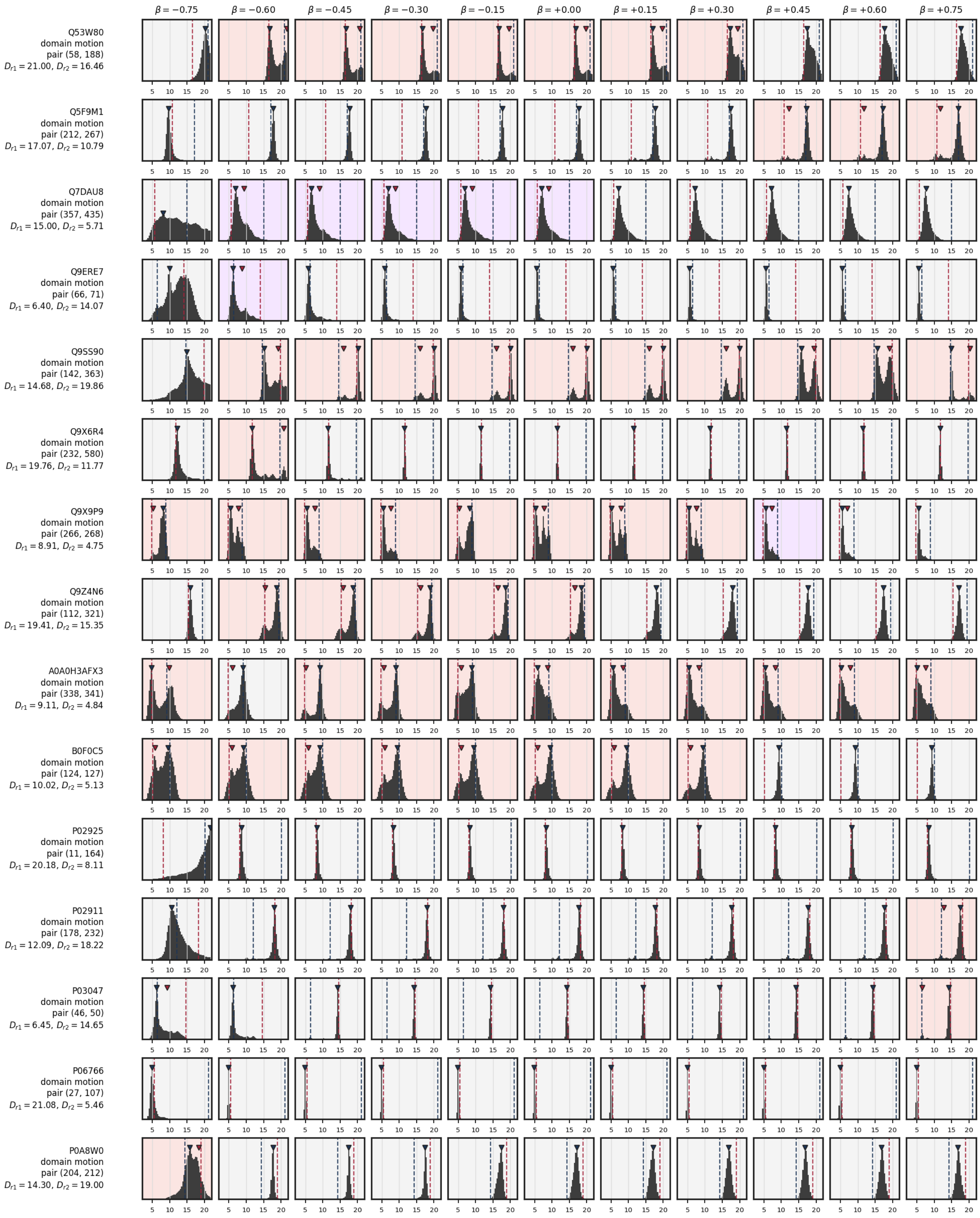

Figure S12. Per-target distogram bimodality, page 2 of 6. Panels as in Figure S11.

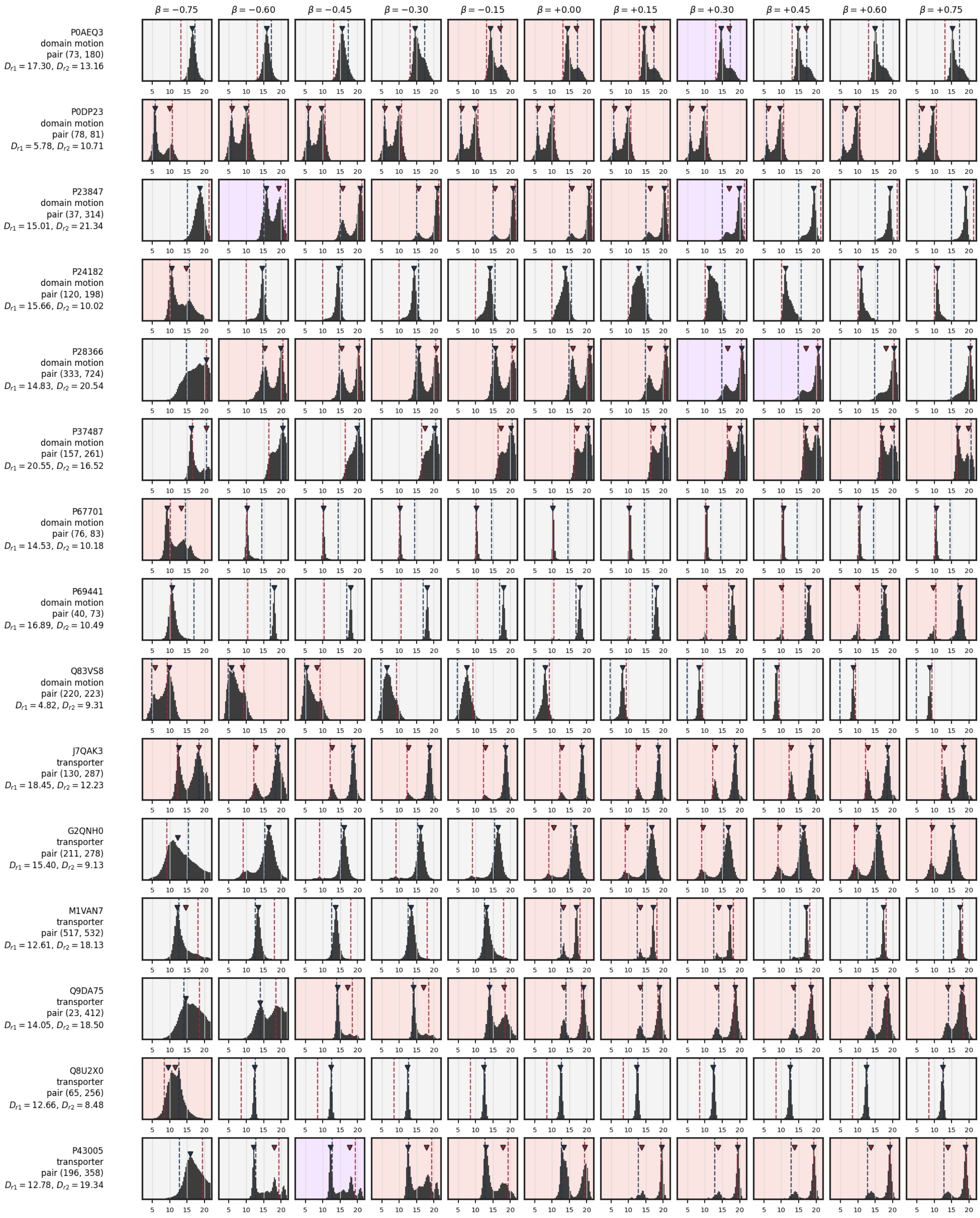

Figure S13. Per-target distogram bimodality, page 3 of 6. Panels as in Figure S11.

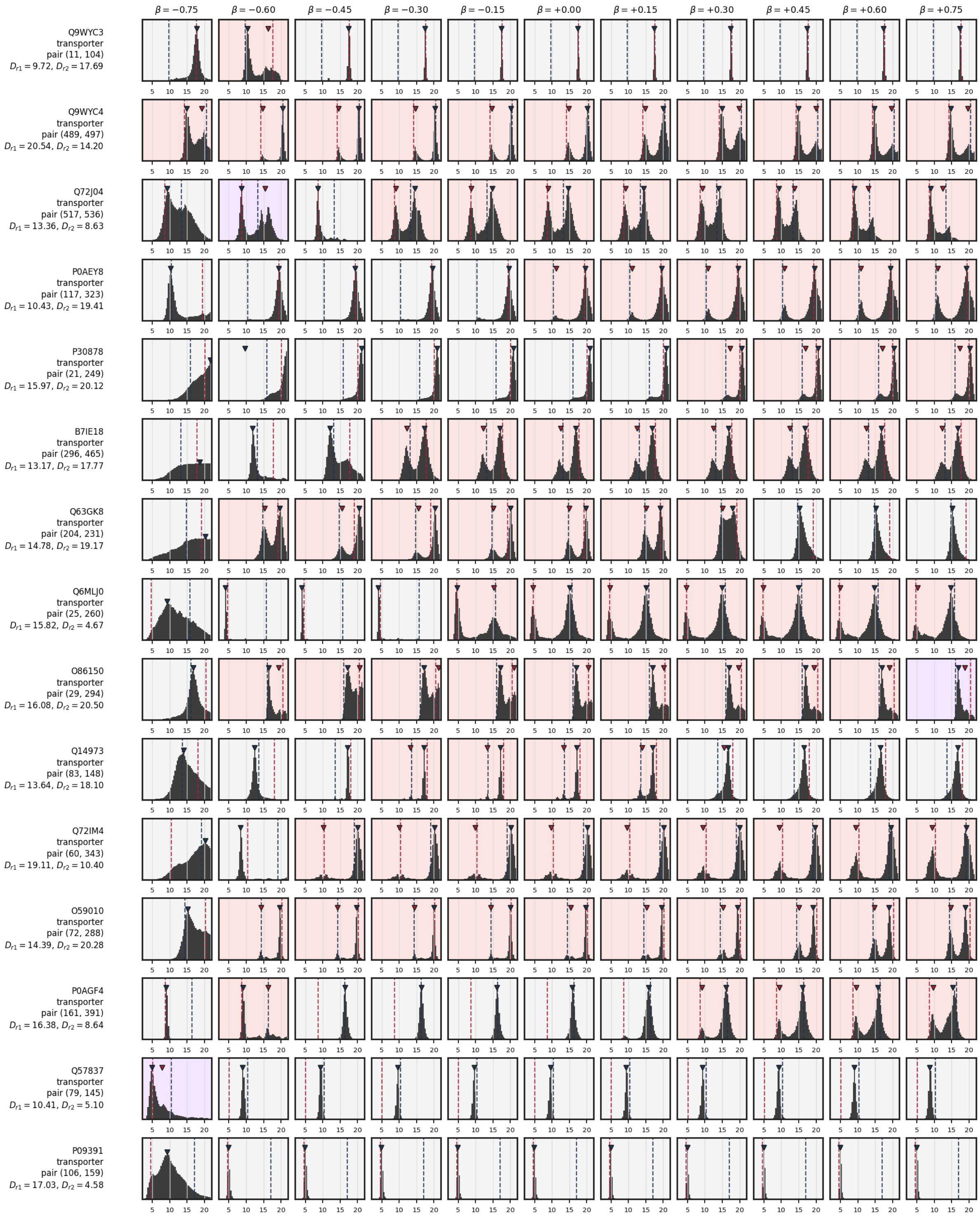

Figure S14. Per-target distogram bimodality, page 4 of 6. Panels as in Figure S11.

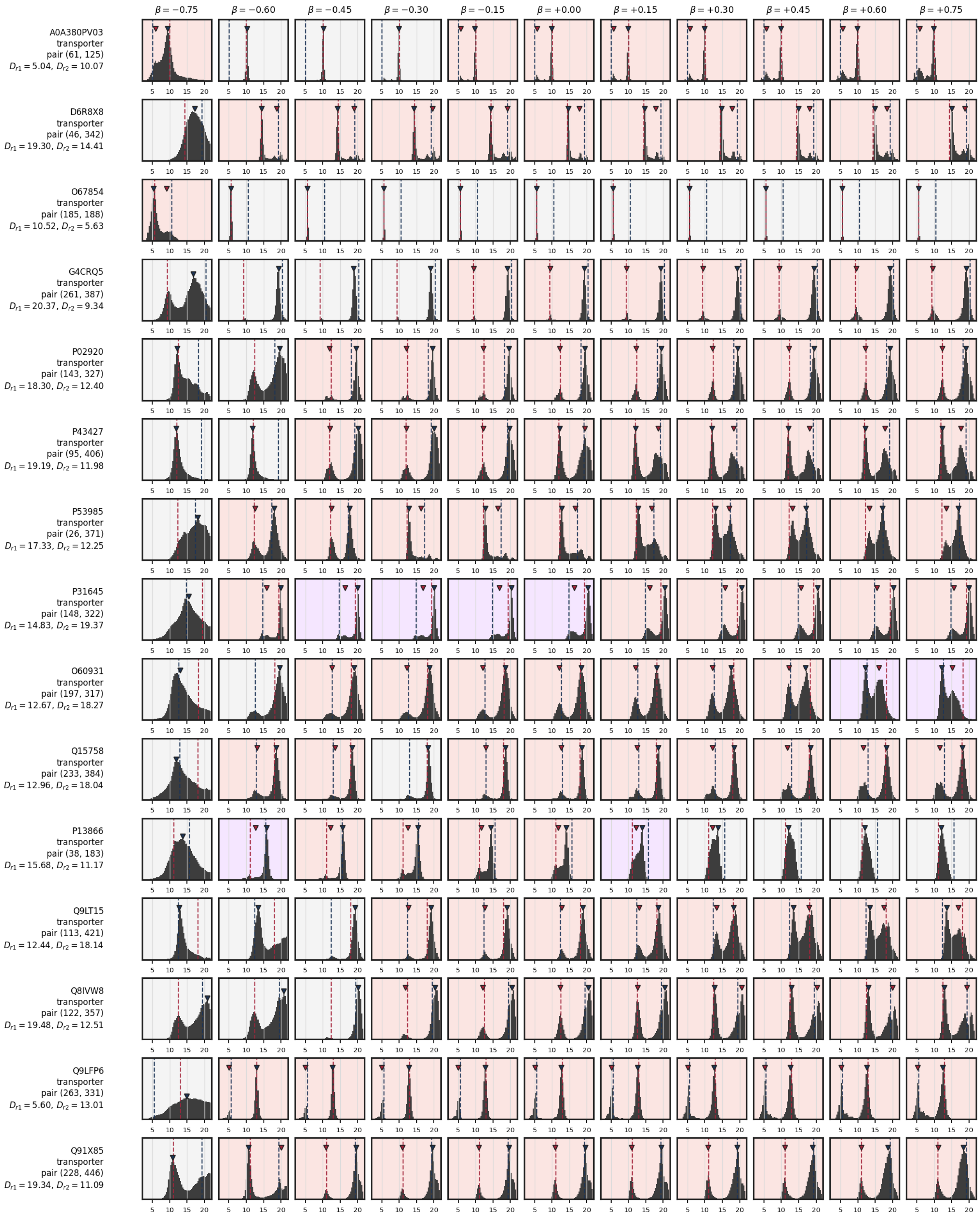

Figure S15. Per-target distogram bimodality, page 5 of 6. Panels as in Figure S11.

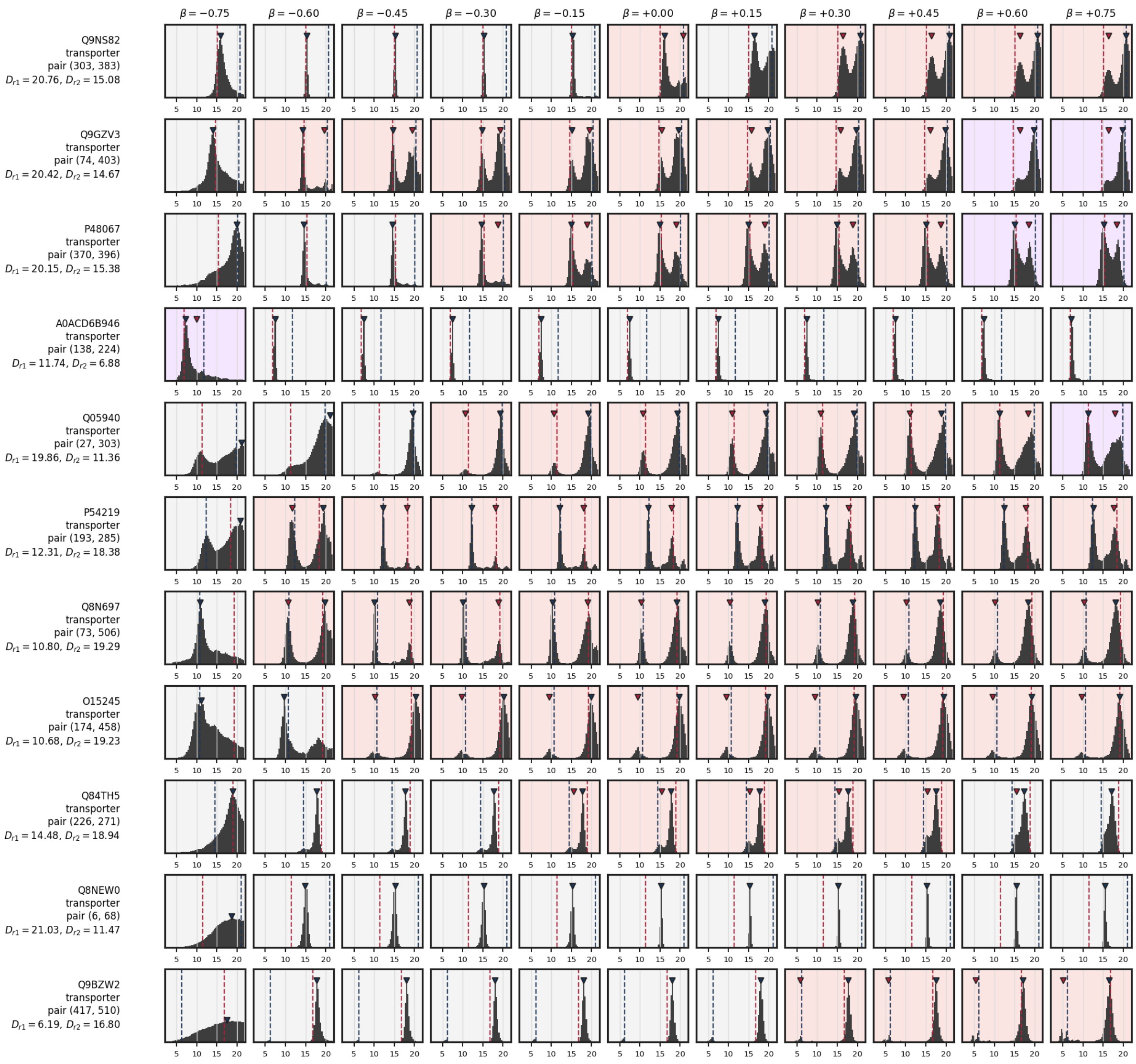

Figure S16. Per-target distogram bimodality, page 6 of 6. Panels as in Figure S11.
